# More Filtering on SNP Calling Does Not Remove Evidence of Inter-Nucleus Recombination in Dikaryotic Arbuscular Mycorrhizal Fungi

**DOI:** 10.3389/fpls.2020.00912

**Published:** 2020-07-07

**Authors:** Eric C. H. Chen, Stephanie Mathieu, Anne Hoffrichter, Jeanne Ropars, Steven Dreissig, Jörg Fuchs, Andreas Brachmann, Nicolas Corradi

**Affiliations:** ^1^Department of Biology, University of Ottawa, Ottawa, ON, Canada; ^2^Genetics, Biocenter, LMU Munich, Martinsried, Germany; ^3^Ecologie Systématique Evolution, CNRS, AgroParisTech, Université, Paris-Saclay, Paris, France; ^4^Leibniz Institute of Plant Genetics and Crop Plant Research, Gatersleben, Germany

**Keywords:** recombination and evolution, single nucleus sequencing, parasexuality, dikaryosis, arbuscular mycorrhizal fungi

## Abstract

Evidence for the existence of dikaryote-like strains, low nuclear sequence diversity and inter-nuclear recombination in arbuscular mycorrhizal fungi has been recently reported based on single nucleus sequencing data. Here, we aimed to support evidence of inter-nuclear recombination using an approach that filters SNP calls more conservatively, keeping only positions that are exclusively single copy and homozygous, and with at least five reads supporting a given SNP. This methodology recovers hundreds of putative inter-nucleus recombination events across publicly available sequence data from individual nuclei. Challenges related to the acquisition and analysis of sequence data from individual nuclei are highlighted and discussed, and ways to address these issues in future studies are presented.

## Introduction

Genome-based analyses have uncovered a large number of signatures of sexual reproduction in the arbuscular mycorrhizal fungi (AMF), challenging the notion that these organisms are ancient asexuals (Halary et al., 2011, 2013; Tisserant et al., 2013; Riley et al., 2014; Corradi and Brachmann, 2016; Ropars et al., 2016; Chen et al., 2018a). Notably, genome analyses showed that model AMF in the genus *Rhizophagus* can be either homokaryotic, carrying thousands of nuclei with a similar genotype, or dikaryotic, whereby nuclei from two parental genotypes are continuously present in the cytoplasm.

Furthermore, the nuclei of dikaryotic AMF isolates each carry one of two divergent regions that resemble the mating-type (MAT) loci of sexual fungi – i.e., putative idiomorphs. The MAT-locus is a genomic region that governs sexual identity in fungi (Fraser and Heitman, 2003; Heitman et al., 2013). In dikaryotic sexual fungi, co-existing nuclei are expected to recombine either through sex (Lee et al., 2010; Heitman, 2015) or somatic events (Xu et al., 1996; Clark and Anderson, 2004; Anderson and Kohn, 2007; also see review from Yildirir et al., 2020). To confirm the existence of low nuclear diversity and dikaryotic stages in AMF, as well as to test whether recombination occurs among co-existing nuclei, a recent study sequenced 86 single nuclei from seven AMF isolates (Chen et al., 2018b). This study supported the hypothesis that two genotypes co-exist in some AMF isolates, and confirmed that overall nuclear genetic diversity is low in these organisms. Remarkably, it also showed evidence that rare inter-nucleus recombination events can be found in dikaryotic AMF strains (Chen et al., 2018b).

The discovery of inter-nuclear recombination in AMF was, however, challenged. Specifically, it was suggested that recombination events in AMF drop significantly once heterozygous, duplicated regions covering SNPs and sites supported by less than five reads are removed from available datasets from single nuclei (Auxier and Bazzicalupo, 2019). Here, we show that the removal of duplicates, heterozygous positions and sites supported by less than five reads still retrieves significant inter-nuclear recombination within available datasets (Chen et al., 2018b). Lastly, we find little support that recombinant sites identified along low coverage regions (Chen et al., 2018b) are artifactual.

## Results

### Hundreds of Cases Involving Inter-Nucleus Recombination Are Retrieved Using Stringent Filtering in Dikaryotic Isolates of *Rhizophagus Irregularis*

Reports of rare inter-nucleus recombination in AMF (Chen et al., 2018b) were based on the analysis of downstream and upstream regions surrounding SNPs, and present in either one or two copies in AMF dikaryotic genome assemblies. Heterozygous sites were also removed in that study, with the exception of genotypes carrying heterozygosity that were identical to those found in homozygous nuclei. Finally, the abovementioned study also analyzed all sites with a coverage of 2 or higher.

Here, we implement a more conservative approach for analyzing the same single nucleus genome datasets, which are always noisy. Because conservative methods can be applied to study larger datasets, we implemented it to a larger dataset – i.e., 1000 contiguous, as opposed to 100 analyzed in Chen et al. (2018b) - to gather a better view of recombination events in AMF dikaryons. The method focuses on sites with a coverage >5, and removes duplicates and nuclei with heterozygous positions. This method allowed us to search for putative inter-nuclear recombination events along 37 to 50% of the three dikaryotic reference genomes SL1, A4, and A5 (Note that the average assembly coverage for single nuclei Illumina read varies from 11% for SL1 to 58% for A5).

We mapped reads from single nuclei against their corresponding dikaryotic reference genomes (e.g., single nuclei reads from SL1 against SL1 reference genome, etc.) and scored SNPs using Freebayes with following parameters: -p 1 -m 30 -K -q 20 -C 2. We consider as evidence of inter-nucleus recombination cases where: (1) one or two contiguous SNPs match the haplotype carried by nuclei with the opposite MAT locus (a genomic regions putatively involved in sex determination in AMF); and (2) at least three contiguous SNPs match the haplotype carried by nuclei with the opposite MAT locus.

For scenario #1, which was not analyzed by a recent comment on Chen et al., 2018b, we detected a total of 913 cases (SL1:115; A5:193; A4:605). These mutational events are unlikely to represent sequencing errors or somatic mutations, as they always produce the opposite co-existing genotype (as opposed to random, nucleus-specific substitutions). These sites were recovered along single copy, homozygous sites with at least five reads supporting the given SNP position.

For the scenario #2, where variation along individual single nuclei spans more than three contiguous SNPs, our analysis recovered 195 recombinant blocks (SL1: 36; A5: 30; A4: 129; Supplementary Tables S1, S2). Of these, 3, 2, and 7 blocks are, respectively found in the first 100 scaffolds. Remarkably, these recombinant blocks include up to 17 contiguous SNPs, and between 172 (in the isolate A5) to 635 (in the isolate A4) SNPs in total. Blocks can encompass up to 7 kb of individual nuclear genomes (Supplementary Tables S1, S2).

This re-analysis of the original dataset published in Chen et al. (2018b) shows that using more stringent filters, i.e., single copy and homozygous sites with at least five reads supporting a SNP, does not remove evidence of inter-nuclear recombination. It also confirms that putative inter-nuclear recombination is indeed a rare event in AMF dikaryons, as was originally reported by Chen et al. (2018b) Note that the report of inter-nuclear recombination by Chen et al. (2018b) was based on observed patterns, as opposed to “bin counting.” Furthermore, the higher recombination rates originally observed by Chen et al. (2018b) in the isolate SL1 was primarily based on a more continuous genome assembly obtained using ALLPATHS-LG (Butler et al., 2008). This assembly is not analyzed here to ensure for direct comparisons of recombination events between AMF dikaryotic isolates – i.e., all assemblies analyzed here were obtained using SPades (Bankevich et al., 2012).

### Recombination in Low Coverage and High-Coverage Sites

Wide read coverage variability is a hallmark of all single nuclei sequencing studies, as this method relies on DNA amplification procedures such as multiple amplification displacement (MDA) to improve yield. As a result of this variability, low coverage calls - i.e., positions supported by less than five reads - represent 35 to 54% of available single nucleus data from dikaryotic isolates. Evidently, a very significant amount of sequence data from AMF single nuclei is located in regions with low coverage.

The application of best practices in genome analysis − e.g., the removal of low coverage positions – are key steps to improve SNP validation in uniformly covered genome references. However, such practices also automatically eradicate a significant amount of data from single nucleus sequencing projects. This begs the question; are low coverage SNP calls identified along single nucleus data mostly untrustworthy and random? Our data suggests that they are not.

This view is supported by data available for the two dikaryotic isolates A4 and A5. Specifically, the genotypes of single nuclei isolated from these two isolates were mainly produced from positions with low read depth (Auxier and Bazzicalupo, 2019). However, despite the overabundance of low coverage sites, the 150 base pairs paired-end Illumina reads from single nuclei still did their job well, mapping with high fidelity to their genome assemblies to generate a clear dikaryotic patterns for these isolates (see Figure 2a,b in Chen et al., 2018b). In both cases, each genotype is linked with a specific MAT-locus (Chen et al., 2018b); a dichotomy that should not emerge if the abundant low coverage sites produced mostly false SNP calls in dikaryotic isolates.

**FIGURE 1:**
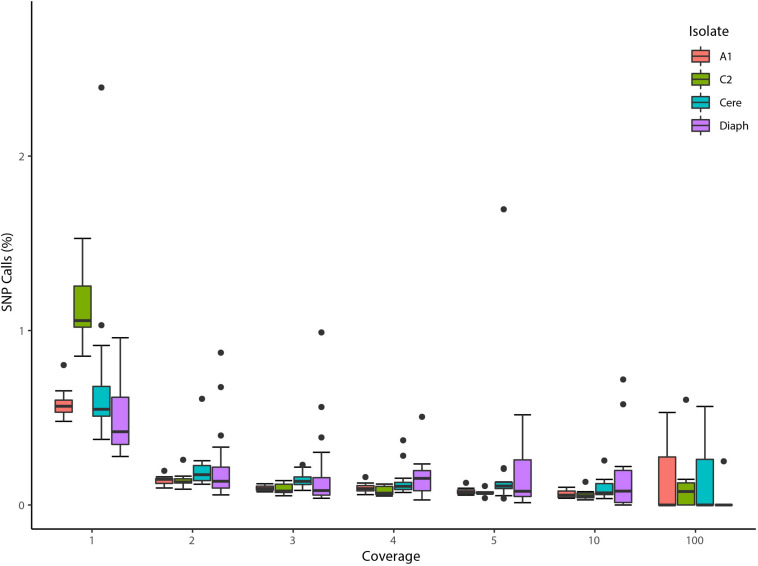
Validation of low coverage depth SNP calls based on single nuclei Illumina reads from four homokaryotic strains. The boxplot represents the percentage of SNPs found to be in disagreement with reference assemblies in two *R. irregularis* isolates (A1 and C2), *R. cerebriforme* (Cere), and *R. diaphanous* (Diaph) organized by the number of reads supporting a given SNP. Boxes represent 25–75% percentile and whiskers represent the largest and smallest value within 1.5 interquartile range above 75th or below 25th percentile. One outlier from *R. diaphanus* is not shown (SN17, coverage 100: 1 mismatch out of 36 positions).

Consistent with this, we found that low coverage reads in 5 homokaryotic isolates (isolates A1, C2, B3 belonging to *Rhizophagus irregularis* species, *Rhizophagus diaphanus* - MUCL-43196, and *R. cerebriforme* - DAOM227022) produce high quality reference genotypes >99.985% of the time, regardless of size and fragmentation of the assembly (Figure 1). Note that the high accuracy of these calls is virtually identical to those made with much larger coverage - i.e., from 5 to 100 (99.987%).

To further analyze low coverage calls in dikaryotic strains, we investigated if those could validate a dikaryotic genotype found years earlier using PCR and Sanger sequencing (Ropars et al., 2016). These genotypes were originally identified from the A5 scaffolds 2, 17, 37, and 641 (see columns representing individual nuclei from A5 in Figure 3 of Ropars et al., 2016). Remarkably, by implementing the same read mapping method used by Chen et al. (2018b), the exact genotypes originally found by Ropars et al. (2016) are fully recovered using the paired-end nucleus Illumina reads from A5. In all cases, the genotypes are linked with their respective MAT-locus and, notably, all positions (16/16) with a read depth ranging from 1 to 4 produce the expected genotype (Supplementary Table S3), further supporting the notion that the 150 paired-end reads can distinguish nucleus-specific homologous single copy regions.

We also aimed to determine if positions supported by two to four reads would result in a dramatic increase in recombination events. Again, this would be expected if these SNP calls were mainly spurious. To do this, we sought evidence of inter-nucleus recombination along single copy, homozygous regions with minimum two reads supporting a SNP position. This analysis retrieved, respectively, 73, 168, and 31 recombinant blocks in SL1, A4 and A5, ranging from a minimum of 3 to a maximum of 21 contiguous SNPs (Supplementary Tables S4, S5). Overall, the number of recombinant blocks supported by less than five reads varies, but does nevertheless remains within the same range – e.g., the number of blocks increases by 3% for A5 and 23% for A4). The larger block number increase seen in SL1 (36 to 73) simply reflects the low nucleus-specific coverage of this isolate, which results from a genome reference that has more than twice the number of contigs compared to other dikaryotic isolates, despite being generated with longer mate-pair libraries and higher coverage. In our view, the unique high genome fragmentation of SL1 indicates the higher genetic complexity of this isolate – e.g., higher recombination rates? (Supplementary Table S1).

**FIGURE 2:**
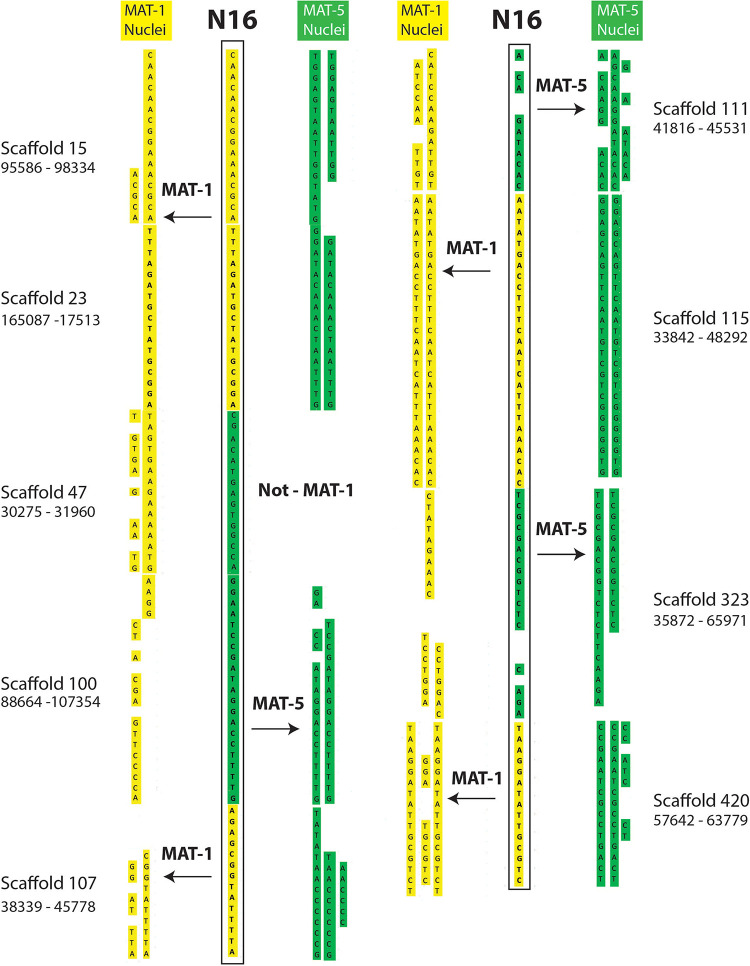
Examples of inter-nucleus recombination in a nucleus of the dikaryotic isolate SL1. The regions are found along homozygous regions present only once in the reference genome of SL1. The nucleus 16 of SL1 carries a genotype that is overwhelmingly similar to nuclei carrying the MAT-1 locus (yellow). In several instances, however, the SN16 is found to switch alleles to carry the other co-existing genotype (green) over several kilobases.

**FIGURE 3:**
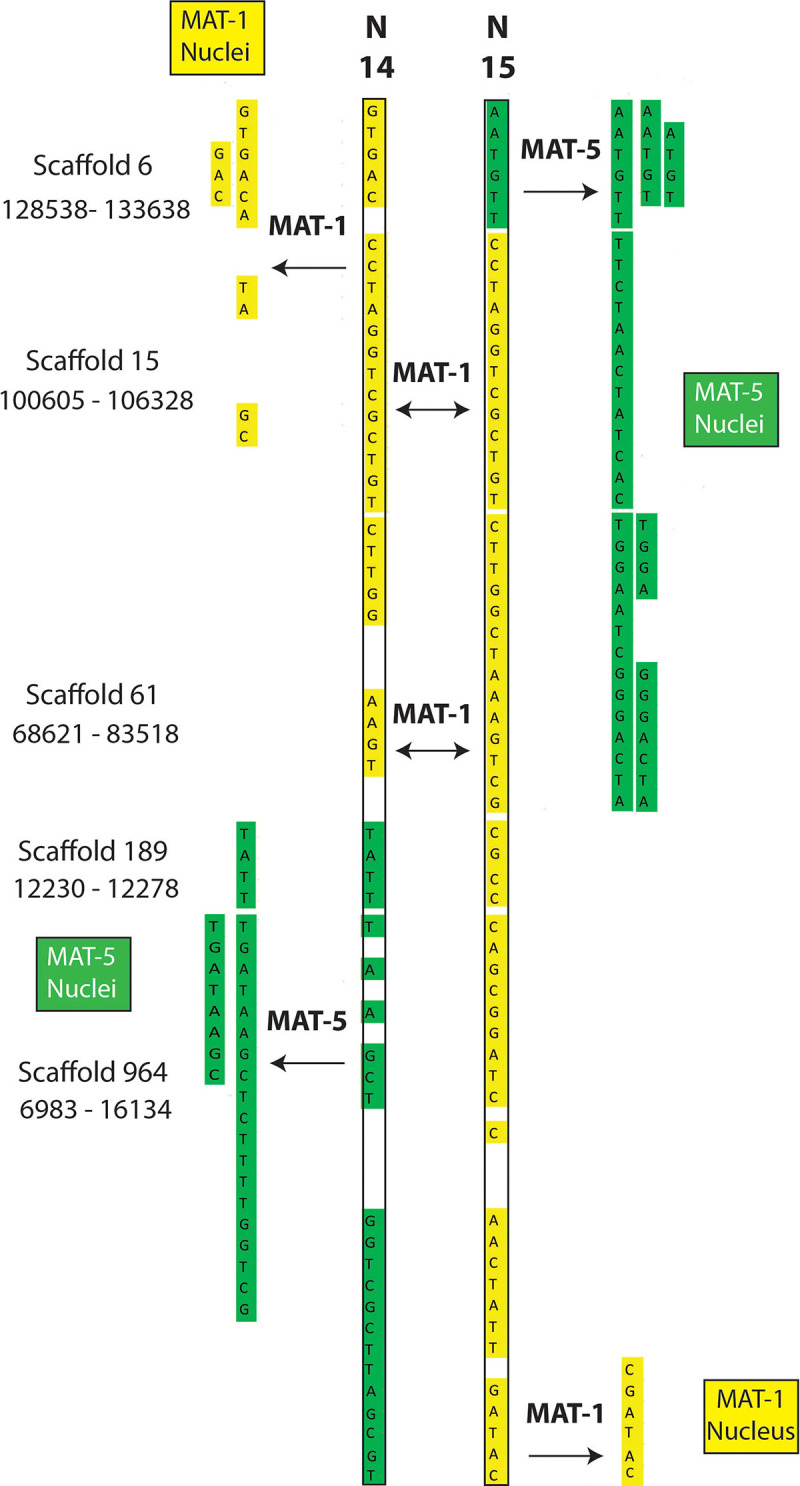
Examples of recombination involving two nuclei (SN14 and SN15) of the dikaryotic isolate SL1. The regions are found along homozygous regions present only once in the reference genome of SL1. The nuclei N14 and 15 from SL1 carry the MAT-1 locus (validated by PCR) and, accordingly, their sequenced genotypes are almost identical. In some cases, however, each nucleus swaps genotype with the opposite MAT locus (i.e., genotype becomes green).

The putative recombination events found along regions supported by less than 5 reads (Supplementary Tables S4, S5) include cases where single nuclei swap genotypes back and forth along up to 60 Kb (Figures 2, 3); something that is difficult to explain based on low coverage alone. Lastly, we find that many recombinant sites are also found in single copy regions with read coverage much higher than five (see a few examples in Supplementary Table S6). As such, evidence of inter-nucleus recombination in available single nucleus sequence datasets is also supported by very high coverage data along single copy regions.

Overall, the wide coverage of heterogeneity characteristic of individual nuclei sequencing clearly makes the application of best practices in genome analysis difficult – e.g., >50% of the data could be removed solely based on coverage threshold of five alone, despite evidence that most of these sites of high quality. Therefore, even though such practice should always be applied to study data from individual nuclei to support their genotyping, low coverage data should not be completely dismissed *a priori* and may be best analyzed on a case-by-case basis for quality.

### Confirming That Nucleus 07 (SL1) Carries the MAT-5 Locus Would Be Compelling Evidence Against Recombination in This Nucleus

Using PCR and sequencing, Chen et al. (2018b) found that Nucleus 07 of SL1 (SL1_07) carries the MAT-locus 1, even though its nuclear genotype often resembles that of co-existing MAT-5 nuclei. It was noted that Illumina reads covering the MAT-locus 1 are not present in the in SL1_07 single nucleus data, and it was thus suggested that this specific sample may have been mixed-up (Auxier and Bazzicalupo, 2019).

Interestingly, we also note that: (1) Illumina reads that map the MAT-locus 5 are also absent from the SL1_07 data, even though their presence would provide compelling evidence against recombination in this nucleus and support of sample mix-up; (2) Other nuclei have no evidence of read mapping along the MAT-locus and all still had their MAT-locus identity properly confirmed by PCR/Sanger like SL1_07; (3) SL1_07 carries substantial evidence of recombination beyond the MAT-locus, particularly in the ALLPaths-LG assembly [see Supplementary file 7 in Chen et al. (2018b)].

Clearly, the absence of sequencing reads covering the MAT-locus provides no evidence against the presence of recombination in the nucleus SL1_07.

## Conclusion

The aim of Chen et al. (2018b) was not to make an inventory of inter-nucleus recombination events in AMF but rather to:

(a) Validate the existence of a unique dikaryotic condition in some AMF isolates, whereby several thousands of nuclei that are copies of two parental genotypes co-exist in one large cell following plasmogamy between compatible homokaryons.
(b) Identify the degree of nuclear diversity within the AMF mycelium, which is found to be always low in the genus *Rhizophagus.*
(c) Detect evidence of inter-nucleus recombination in three dikaryotic isolates.

A recent comment on the single nucleus analyses recently published focused exclusively on point (c). Yet, by re-analyzing the same single nucleus data with more stringent filters (single copy and homozygous sites with more than five reads supporting a SNP), we still find that dikaryotic isolates carry significant evidence of inter-nucleus genetic exchange.

At a minimum, these findings confirm what is already known – i.e., co-existing nuclei in conventional dikaryotic cells (2 nuclei/cell) show footprints of recombination similar to those observed here (Clark and Anderson, 2004; Anderson and Kohn, 2007; Yildirir et al., 2020). As such, to suggest that AMF do not undergo similar processes, one must assume that millions of nuclei from two parental genotypes can co-exist in the same cytoplasm for decades without undergoing genetic interactions.

Despite present findings, it is fitting to end on a cautionary note regarding the use of read mapping to genotype individual nuclei. Specifically, even though the present work validates previous findings (identification of dikaryotic genotypes, low diversity) and the methodology we used is appropriate to test inter-nuclear recombination, the work of Chen et al. (2018b) also relies on sequence data that can vary dramatically in terms of coverage and quality [as a result, for example, of multiple displacements during genome amplification, PCR bias during Illumina sequencing, or rare DNA cross-contamination (Dreissig et al., 2015, 2017)]. It also relies on a reference genome and pre-determined mapping thresholds that can all independently affect the analysis output.

Thus, like for any biological finding, it will be important for future studies to validate the presence of inter-nucleus recombination using alternative methods. To this end, plans are underway to sequence individual AMF nuclei using long-read sequencing technologies, and perform single nuclei genotyping using complete, phased genome references for all dikaryotic AMF isolates. Long-read sequencing will be important to reveal the exact origin of the heterozygous sites in single nuclei datasets – i.e., whether some of these represent miss-mapped reads, sequence errors, aneuploidy or contaminants - as these are often found along recombination tracts (see paper and Auxier and Bazzicalupo, 2019). Lastly, producing a recombining progeny by crossing compatible homokaryotic AMF isolates will be key to conclusively demonstrate how/when sexual reproduction (meiosis) occurs in AMF.

## Materials and Methods

### Obtaining Genotype Files

For filtering and generating genotype files in Supplementary Tables S2, S5, the original method described in Chen et al. (2018b) was used with three modifications. First, the number of BLAST hits allowed is reduced to just one (from two) so that no duplicated region is taken into account. The second modification relates to the treatment of heterozygosity. Sites for individual nuclei that did not pass the 10-to-1 alternate to reference allele test based on Freebayes (Garrison and Marth, 2012). SNP caller (hence forth referred to as “10-to-1”) are now removed, even in cases where their genotype is confirmed by homozygous nuclei, which was the approach originally used in Chen et al. (2018b). The final modification is extending the number of scaffolds surveyed to first 1000 scaffolds (from 100).

### Homokaryon Low-Coverage Read Analysis

To assess the fidelity of low coverage calls (Figure 1), homokaryon isolate A1, C2, *R. cerebriforme*, and *R. diaphanus*, are used. From the mapped BAM file of each single nucleus sequencing, we extracted positions from the first 10 scaffolds whose position have coverage of 1, 2, 3, 4, 5, 10, and 100. For each nucleus, positions with indel calls are filtered out. The 10-to-1 is also used on heterozygous positions. Finally, the percentage of homozygous mismatches are then calculated and collected across nuclei of each isolate before plotting in R via ggplot2, reshape2, grid, and grid_extra.

The difference in the number of recombined blocks identified in our study and a recent challenge may be a consequence of the treatment of heterozygous sites. Specifically, in the recent challenge to Chen et al. (2018b), it appears (based on their script) that the presence of heterozygosity in one nucleus (something that can be created by a single miss-mapped read) immediately results in complete removal of all homologous sites from all other co-exiting nuclei, regardless of whether these sites are homozygous and with high coverage. This approach drastically reduces opportunities to compare *bona-fide* genotypes/blocks.

In contrast to this, in our methodology the heterozygous nuclei – i.e., potential artifactual recombinants - are completely removed and not analyzed, but the co-existing nuclei with homologous homozygous and high coverage positions are kept for downstream analyses.

### Genotype Identity

The goal of color labels is to make it easier the observation of recombination footprints between nuclei. It is not to produce complete haplotypes. We assign genotype color first based on parsimony using nuclei with PCR validated mating type: the mating type with more nuclei showing a particular genotype gets a color assigned. If it is a tie, or there is no PCR-proven mating type, then color that does not suggest new recombination is assigned (no change of color down the column). If that fails, first genotype in that row to be *MAT*-A. The exception is SL1’s nucleus SN07 where in tied situations the color corresponding to *MAT*-5 is assigned, which is consistent with its genotype clustering with other *MAT*-5 nuclei (Chen et al., 2018b).

To score recombination events in scenario #1, a site is flagged as “recombining” if it starts to share one of two consecutive SNP with nuclei of the other MAT-locus along the same scaffold. For scenario #2, the same process is used, but a minimum of 3 consecutive SNP must be present.

For both scenarios, we count the number of events in each nucleus. For example, if 2 nuclei show recombination at the same location, the total number of events identified would be 2. Finally, in SL1’s SN07, we sometimes manually correct the coloring to highlight instances where it did not have recombination. This is purely for clarity only and does not affect the counting of recombination events.

### Obtaining Read Support of Each Position

To generate the read support for Figure 1 and Supplementary Tables S1–S6, we opt to use bam-readcount^1^ (version 0.8.0). We used the original bam files from Chen et al. (2018b). and queries for positions of interest. In the reanalysis of genotypes from Ropars et al., 2016, we used BLAST to identify the location of PCR products.

## Data Availability Statement

Publicly available datasets were analyzed in this study. This data can be found here: ID LLXH00000000, LLXI00000000, LLXJ00000000, LLXK00000000, LLXL00000000, and PRJNA477348.

## Author Contributions

EC: hypothesis, formal analysis, validation, investigation, visualization, methodology, and writing–review and editing. SM: validation, investigation, and visualization. AH: formal analysis, validation, investigation, visualization, methodology, and writing–review and editing. JR: review and editing, and hypothesis. JF, SD, and AB: review and editing. NC: writing, hypothesis, and visualization. All authors contributed to the article and approved the submitted version.

## Conflict of Interest

The authors declare that the research was conducted in the absence of any commercial or financial relationships that could be construed as a potential conflict of interest.

## Footnotes

1 https://github.com/genome/bam-readcount

## Supplementary Material

TABLE S1 Genotype file with minimum coverage of 5, with heterozygous sites as defined in Chen et al. (2018b) and Auxier and Bazzicalupo (2019) removed, and single-copy regions. Yellow and Green colors highlight the two co-existing genotypes. Nuclei highlighted in yellow and green had their MAT locus validated by PCR. Nuclei with cells highlighted in light green carry a genotype that is mostly associated with green nuclei validated by PCR, while those with cells highlighted in orange carry a genotype that is mostly associated with yellow nuclei validated by PCR.

TABLE S2 Recombination blocks (Scenario #2) obtained from genotype file with at least five reads supporting a SNP in the first 1000 contiguous. Based on Supplementary Table S1.

TABLE S3 Confirmation of genotypes found by Ropars et al., 2016 Nature Microbiology using PCR and Sanger sequencing. F (A) = = Reads supporting genotype (reads against genotype). Read depth is shown on the left columns for each position. Numbers in bold represent valid genotypes with very low read depth, i.e., 1 to 5.

TABLE S4 Recombination blocks (Scenario #2) obtained from genotype file with at least two reads supporting a SNP in the first 1000 contiguous. Based on Supplementary Table S5.

TABLE S5 Genotype file with minimum coverage of 2, with heterozygous sites as defined in Chen et al. (2018b) and Auxier and Bazzicalupo (2019) removed, and based on regions found only once in the reference genome. Yellow and Green colors highlight the two co-existing genotypes. Nuclei highlighted in yellow and green in had their mating-type locus validated by PCR. Nuclei with cells highlighted in light green carry a genotype that is mostly associated with green nuclei validated by PCR, while those with cells highlighted in orange carry a genotype that is mostly associated with yellow nuclei validated by PCR.

TABLE S6 Examples of recombination with high coverage. Nuclei ID are colored for identification purposes. Yellow and dark green *MAT*-locus that is PCR verified. Yellow and Green colors highlight the two co-existing genotypes. Nuclei highlighted in yellow and green in had their mating-type locus validated by PCR. Nuclei with cells highlighted in light green carry a genotype that is mostly associated with green nuclei validated by PCR, while those with cells highlighted in orange carry a genotype that is mostly associated with yellow nuclei validated by PCR.

## References

[B1] Anderson J. B. Kohn L. M. (2007). “Dikaryons, diploids, and evolution,” in *Sex in Fungi.* eds Heitman J. Kronstad J. Taylor J. Casselton L. (Washington, DC: ASM Press), 333–348. 10.1128/9781555815837.ch20

[B2] Auxier B. Bazzicalupo A. (2019). Comment on “single nucleus sequencing reveals evidence of inter-nucleus recombination in arbuscular mycorrhizal fungi”. *eLife* 8 1–9. 10.7554/eLife.47301 31650958 PMC6814362

[B3] Bankevich A. Nurk S. Antipov D. Gurevich A. A. Dvorkin M. Kulikov A. S. (2012). SPAdes: a new genome assembly algorithm and its applications to single-cell sequencing. *J. Comput. Biol.* 19 455–477. 10.1089/cmb.2012.0021 22506599 PMC3342519

[B4] Butler J. MacCallum I. Kleber M. Shlyakhter I. A. Belmonte M. K. Lander E. S. (2008). ALLPATHS: de novo assembly of whole-genome shotgun microreads. *Genome Res.* 18 810–820. 10.1101/gr.7337908 18340039 PMC2336810

[B5] Chen E. C. H. Mathieu S. Hoffrichter A. Ropars J. Dreissig S. (2020). More filtering on SNP calling does not remove evidence of inter- nucleus recombination in dikaryotic arbuscular mycorrhizal fungi. *bioRxiv [Preprint]* 10.1101/2020.01.15.906412 PMC7358544 32733503

[B6] Chen E. C. H. Morin E. Beaudet D. Noel J. Yildirir G. Ndikumana S. (2018a). High intraspecific genome diversity in the model arbuscular mycorrhizal symbiont *Rhizophagus irregularis*. *New Phytol.* 220 1161–1171. 10.1111/nph.14989 29355972

[B7] Chen E. C. Mathieu S. Hoffrichter A. Sedzielewska-Toro K. Peart M. Pelin A. (2018b). Single nucleus sequencing reveals evidence of inter-nucleus recombination in arbuscular mycorrhizal fungi. *eLife* 7 1–17. 10.7554/elife.39813 30516133 PMC6281316

[B8] Clark T. A. Anderson J. B. (2004). Dikaryons of the basidiomycete fungus *Schizophyllum* commune: evolution in long-term culture. *Genetics* 167 1663–1675. 10.1534/genetics.104.027235 15342506 PMC1470993

[B9] Corradi N. Brachmann A. (2016). Fungal mating in the most widespread plant symbionts? *Trends Plant Sci.* 22 175–183. 10.1016/j.tplants.2016.10.010 27876487

[B10] Dreissig S. Fuchs J. Cápal P. Kettles N. Byrne E. Houben A. (2015). Measuring meiotic crossovers via multi-locus genotyping of single pollen grains in barley. *PLoS One* 10:e0137677. 10.1371/journal.pone.0137677 26356084 PMC4565660

[B11] Dreissig S. Fuchs J. Himmelbach A. Mascher M. Houben A. (2017). Sequencing of single pollen nuclei reveals meiotic recombination events at megabase resolution and circumvents segregation distortion caused by postmeiotic processes. *Front. Plant Sci.* 8:1620. 10.3389/fpls.2017.01620 29018459 PMC5623100

[B12] Fraser J. A. Heitman J. (2003). Fungal mating-type loci. *Curr. Biol.* 13 R792–R795. 10.1016/j.cub.2003.09.046 14561417

[B13] Garrison E. Marth G. (2012). Haplotype-based variant detection from short-read sequencing. *arXiv [Preprint]* Available online at: http://arxiv.org/abs/1207.3907v2 (accessed May 15, 2020).

[B14] Halary S. Daubois L. Terrat Y. Ellenberger S. Wöstemeyer J. Hijri M. (2013). Mating type gene homologues and putative sex pheromone-sensing pathway in arbuscular mycorrhizal fungi, a presumably asexual plant root symbiont. *PLoS One* 8:e80729. 10.1371/journal.pone.0080729 24260466 PMC3834313

[B15] Halary S. Malik S.-B. Lildhar L. Slamovits C. H. Hijri M. Corradi N. (2011). Conserved meiotic machinery in Glomus spp., a putatively ancient asexual fungal lineage. *Genome Biol. Evol.* 3 950–958. 10.1093/gbe/evr089 21876220 PMC3184777

[B16] Heitman J. (2015). Evolution of sexual reproduction: a view from the fungal kingdom supports an evolutionary epoch with sex before sexes. *Fungal Biol. Rev.* 29 108–117. 10.1016/j.fbr.2015.08.002 26834823 PMC4730888

[B17] Heitman J. Sun S. James T. Y. (2013). Evolution of fungal sexual reproduction. *Mycologia* 105 1–27. 10.2307/24637440 23099518

[B18] Lee S. C. Ni M. Li W. J. Shertz C. Heitman J. (2010). The evolution of sex: a perspective from the fungal kingdom. *Microbiol. Mol. Biol. Rev.* 74 298–340. 10.1128/Mmbr.00005-10 20508251 PMC2884414

[B19] Riley R. Charron P. Idnurm A. Farinelli L. Dalpé Y. Martin F. (2014). Extreme diversification of the mating type–high-mobility group (MATA-HMG) gene family in a plant-associated arbuscular mycorrhizal fungus. *New Phytol.* 201 254–268. 10.1111/nph.12462 24033097

[B20] Ropars J. Toro K. S. Noel J. Pelin A. Charron P. Farinelli L. (2016). Evidence for the sexual origin of heterokaryosis in arbuscular mycorrhizal fungi. *Nat. Microbiol.* 1:16033. 10.1038/nmicrobiol.2016.33 27572831

[B21] Tisserant E. Malbreil M. Kuo A. Kohler A. Symeonidi A. Balestrini R. (2013). Genome of an arbuscular mycorrhizal fungus provides insight into the oldest plant symbiosis. *Proc. Natl. Acad. Sci. U. S. A* 110 20117–20122. 10.1073/pnas.1313452110 24277808 PMC3864322

[B22] Xu J. Horgen P. A. Anderson J. B. (1996). Somatic recombination in the cultivated mushroom *Agaricus bisporus*. *Mycol. Res.* 100 188–192. 10.1016/S0953-7562(96)80119-80115

[B23] Yildirir G. Malar C. M. Kokkoris V. Corradi N. (2020). Parasexual and sexual reproduction in arbuscular mycorrhizal fungi: room for both. *Trends Microbiol.* 28 518–520. 10.1016/j.tim.2020.03.013 32360097

